# 170 years of data-mining: history and future

**DOI:** 10.1007/s00417-023-06359-9

**Published:** 2024-01-17

**Authors:** Bernd Kirchhof

**Affiliations:** Center of Opthalmology, University of Koeln, Koeln, Germany

Data is not everything, but without data, everything is nothing [1]. If data were not so essential for progress and development, people would not occasionally try to falsify them (fake news). Access to original data provides us with a solid basis for discussion. However, it is still more difficult to find suitable data in the “biomedical” area. This is the reason review articles are in demand and we have been accessing journals like *Graefe‘s Archives* since 1854. This also explains why we search through index volumes in physical libraries and why we learn to use virtual indexed search algorithms and machines like “Pub-Med” or “Google-Scholar”. Time and access to knowledge resources are identified as the most common barriers to clinical information seeking [2]. And yet, unfortunately, there are still authors whose submissions are not found suitable for publication after review because of “insufficient new information”.

And yet there is the oncologist who no longer practices because he is no longer able to manage the flood of scientific publications down to the contemporary therapy of an individual patient. And he also exists, the colleague who reads several journals, subscribes to them and has them bound, and prepares excerpts for the further training of his scholars.

A solution is at hand. Instead of finding similar articles using keywords, language is vectorized using AI algorithms. In this way, literature can be searched using natural language (natural language processing (NLP)). NLP [3] is a field of artificial intelligence (AI) that enables computers to understand, interpret, and manipulate human language. In NLP-based keyword augmentation, prior knowledge and reasoning techniques are employed to remove irrelevant search results. Some of us certainly appreciate the necessary chatbots such as those from “Bard” (Google), Bing (Microsoft) or Chat GPT (Open AI) for “data mining” on general topics. These chatbots train for example on “Wikipedia”. “Bard” and comrades, however, are unsuitable for the “biomedical” area: firstly, because copyright law prevents access to scientific databases such as PubMed (National Library of Medicine) and secondly, because they generate prose, mostly without a reference list. For the scientific search, suitable chatbots are for example “Elicit” [4], “Scite” [5] and “Consensus” [6]. They depend on free scientific databases (in scite’s case) or have access to paywalled research articles through partnerships with publishers. Chatbots, like “Elicit”, are addressed by natural language to generate reference lists. They implement NLP to remove irrelevant search results. Firms that own large proprietary databases of scientific abstracts and references are now joining [7]. Elsevier (Scopus-AI) [8], Springer Nature and Clarivate are beginning to add AI search functions to their article and book archives [9]. Clarivate integrates the Web of Science. But be careful! AI searches are prone to “hallucinations”. Such childhood illnesses must be taken into account. So far, you can only go back a few years and search in abstracts. By the way, researchers in Germany only have free access to full texts through research institutions (e.g. universities) that have purchased quotas from publishers. Free access to full-text articles would certainly also be helpful for doctors in private practice, i.e. outside of universities.

In the interest of health care, would it not make more sense if the medical associations also entered into partnerships with publishers who would be open to collect infomation from full-text libraries? Then, perhaps the colleague mentioned previously would have the courage to return to patient care in this rapidly developing field of oncology.
